# Nonproteolytic Roles of 19S ATPases in Transcription of CIITApIV Genes

**DOI:** 10.1371/journal.pone.0091200

**Published:** 2014-03-13

**Authors:** Nagini Maganti, Tomika D. Moody, Agnieszka D. Truax, Meghna Thakkar, Alexander M. Spring, Markus W. Germann, Susanna F. Greer

**Affiliations:** 1 Graduate Program in Cell Biology and Immunology, Department of Biology, Georgia State University, Atlanta, Georgia, United States of America; 2 Department of Chemistry, Georgia State University, Atlanta, Georgia, United States of America; Wayne State University, United States of America

## Abstract

Accumulating evidence shows the 26S proteasome is involved in the regulation of gene expression. We and others have demonstrated that proteasome components bind to sites of gene transcription, regulate covalent modifications to histones, and are involved in the assembly of activator complexes in mammalian cells. The mechanisms by which the proteasome influences transcription remain unclear, although prior observations suggest both proteolytic and non-proteolytic activities. Here, we define novel, non-proteolytic, roles for each of the three 19S heterodimers, represented by the 19S ATPases Sug1, S7, and S6a, in mammalian gene expression using the inflammatory gene CIITApIV. These 19S ATPases are recruited to induced CIITApIV promoters and also associate with CIITA coding regions. Additionally, these ATPases interact with elongation factor PTEFb complex members CDK9 and Hexim-1 and with Ser5 phosphorylated RNA Pol II. Both the generation of transcripts from CIITApIV and efficient recruitment of RNA Pol II to CIITApIV are negatively impacted by siRNA mediated knockdown of these 19S ATPases. Together, these results define novel roles for 19S ATPases in mammalian gene expression and indicate roles for these ATPases in promoting transcription processes.

## Introduction

**Figure 1:**
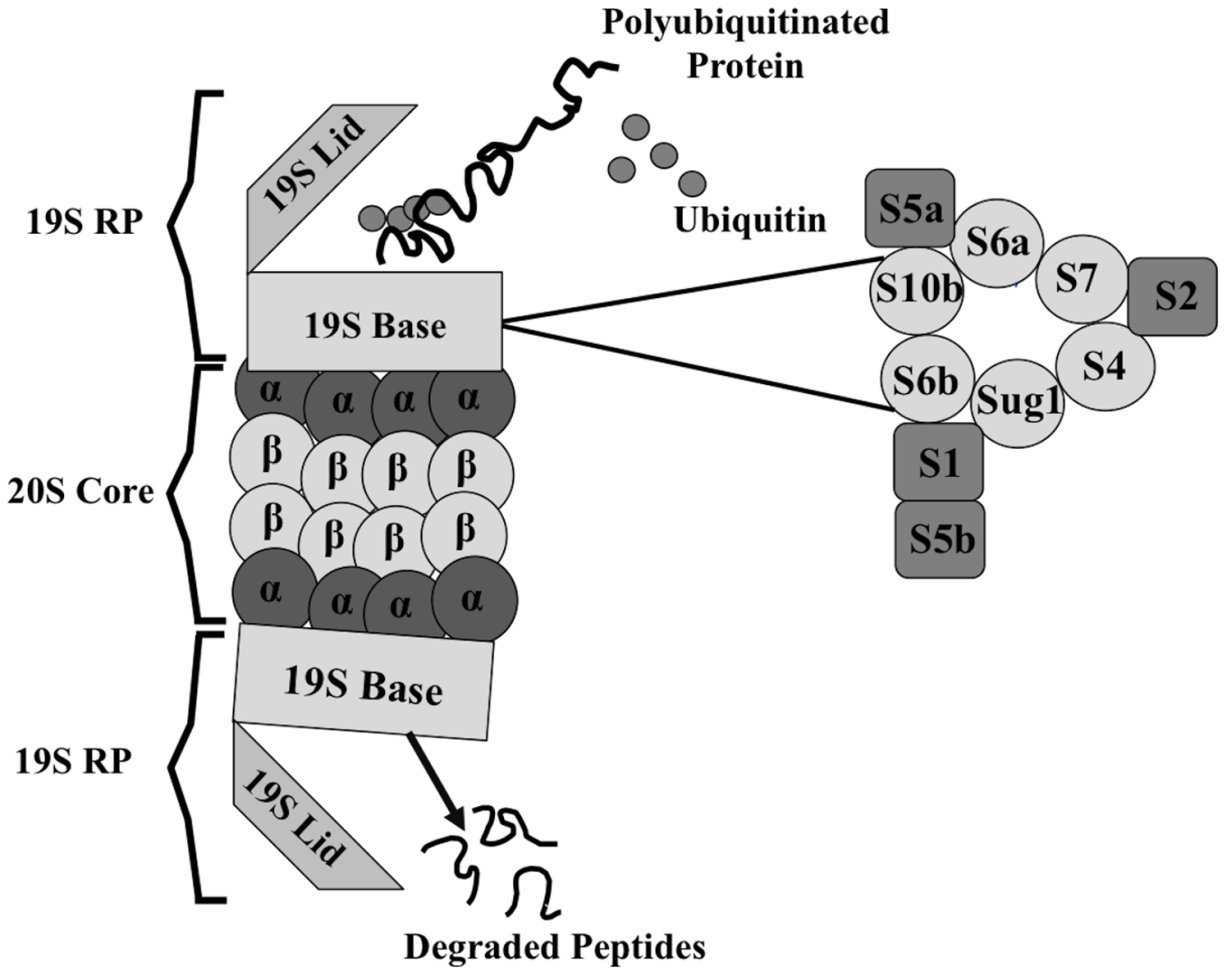
The 26S proteasome is composed of a 20S proteolytic core capped on one or both ends by 19S regulatory particle. The 20S core is a hollow cylindrical structure composed of two heptameric rings of α-subunits and two heptameric rings of β-subunits. The 19S regulatory particle is composed of a base and lid component. The lid component consists of nine non-ATPase subunits and the base is composed of six ATPases (S7, S4, S6a, S10b, Sug1 and S6b) and three non-ATPases (S1, S2, and S5b). Polyubiquitinated proteins are recognized, deubiquitinated, and unfolded by the 19S regulatory particle and the unfolded proteins are translocated to the 20S core where proteins are degraded into small peptides.

Each stage in gene expression involves many proteins that must assemble and disassemble at the right time and place and in the correct order and abundance. While the mechanisms by which cells regulate the location, timing, and amount of proteins involved in gene expression remain unclear, recent observations have linked the 26S proteasome, an essential regulator of protein degradation, to several stages of gene expression. The 26S proteasome in mammalian cells is a 2.5 MDa multi-protein complex comprised of a 19S regulatory particle (RP) and a 20S proteolytic core [1] each of which exists independently in both the nucleus and cytoplasm [2]. The 19S RP is further divided into two parts: a lid and a base. The lid is composed of eight non-ATPase subunits that are required for protein degradation [1], [3], [4]. The base of the 19S contains six ATPases, representing three heterodimeric pairs (Sug1 and S6b, S7 and S4, and S6a and S10b), which belong to the ATPases associated with a variety of cellular activities (AAA) family. The base also contains four non-ATPase subunits: S2, S1, S5a, and S5b [3], [5]–[9]. The 20S catalytic core of the proteasome is a 700 kDa cylinder that consists of four stacked rings, with each ring containing seven α and β subunits [3], [4]. The base ATPases contain a C-terminal hydrophobic tyrosine X motif that docks into the pockets of the α rings of the 20S [10]. In the presence of ATP, the 19S regulatory particle associates with the 20S catalytic core on both sides to form the 26S proteasome, allowing for the recognition of polyubiquitinated substrates marked for degradation [4], [11]. The 19S regulatory particle recognizes the ubiquitin chains on targeted proteins, cleaves the chains, unfolds the protein, and directs the unfolded protein to the 20S core for degradation [4], [12] (Figure 1). Accumulating evidence suggests the 19S proteasome not only recognizes ubiquitinated substrates for proteolysis, but also is linked to gene transcription in numerous different contexts, including mRNA elongation in yeast and mammalian cells [13]–[15].

We detail here non-proteolytic involvement of the 19S ATPases in regulating gene expression from an immunologically important mammalian promoter, the Class II Transactivator (CIITA) which is the master regulator of Major Histocompatibility class II (MHC II) genes [16]. CIITA is expressed constitutively on antigen presenting cells, and is inducibly expressed on all nucleated cells upon stimulation with the inflammatory cytokine interferon gamma (IFN-γ) [17], [18]. CIITA-driven MHC II molecules play critical roles in activating adaptive immune responses by binding and presenting exogenously derived antigenic peptides to CD4^+^ T lymphocytes [16]. MHC II deficiencies lead to the development of Bare Lymphocyte Syndrome (BLS) [19] and Severe Combined Immune Deficiency (SCID) [20] while overexpression of MHC II is associated with the development of autoimmune disease [21]. The presentation of tumor cell antigens by MHC II molecules is critical in the detection of newly formed tumors [22], [23]. Because MHC II molecules play these critical roles in the activation of adaptive immune responses, and since deregulation of MHC II has such dire consequences, MHC II expression is tightly regulated, primarily at the level of transcription by CIITA [24].

Expression of CIITA is regulated in a cell specific manner by four distinct promoters: pI, pII, pIII, and pIV [25]. CIITA expression is induced at pIV when IFN-γ binds to the INF-γ surface receptor [26]–[28]. In addition to promoting the binding of transcription factors to pIV, IFN-γ also induces acetylation of histones, which loosens the chromatin structure and increases the accessibility of CIITApIV [29], [30]. Following IFN-γ stimulation, a large, ubiquitously expressed multi-protein enhanceosome complex, consisting of Regulatory Factor X (RFX), Nuclear Factor Y (NF-Y), and cAMP Response Element Binding (CREB) [31] binds the MHC II proximal promoter and recruits newly expressed CIITA [16], [19]. Once bound to the MHC II enhanceosome, CIITA stabilizes the enhanceosome complex and recruits basal transcription components and RNA Polymerase II (Pol II) [25],[32]–[34].

We recently identified novel roles of the 19S ATPases in regulating acetylation and methylation of histones H3 and H4 at CIITApIV and the MHC II promoter [35]–[38]. Following IFN-γ stimulation, the 19S ATPases bind to the MHC II and CIITApIV promoters. Diminished expression of 19S ATPases inhibits MHC II and CIITApIV promoter histone acetylation and co-factor binding [36], [37], enhances suppressive histone H3 lysine 27 trimethylation [36], and results in repression of transcription [38]. Following chromatin activation by histone modifying enzymes, activator and activator complexes must be recruited to the promoter regions of target genes. To this end, in the absence of 19S ATPases, transcription factor recruitment to CIITApIV and to the MHC II promoter is also dramatically reduced [7], [35]–[37].

Despite growing understanding of the importance of the proteasome in mammalian gene expression, roles for the proteasome in the various stages of transcription remain unclear. We demonstrate here the 19S ATPases have critical, non-proteolytic roles in the regulation of early and intermediate stages of transcription at CIITApIV. The 19S ATPases associate with the CIITApIV proximal promoter, participate in RNA Pol II recruitment, and move into coding regions where they may regulate elongation by their interaction with both inactive and active forms of elongation factor PTEF-b. Depletion of 19S ATPases Sug1, S7, or S6a by siRNA abrogates CIITApIV transcription, with increasing impact on longer transcripts. The 19S ATPases also associate with Ser5p-RNA pol II and their knockdown negatively impacts the recruitment of RNA pol II to CIITA pIV. Together, our studies suggest the 19S ATPases are intimately involved in transcription of the critical adaptive immune gene CIITA.

## Materials and Methods

### Cell Culture

HeLa cells (human epithelial) from ATCC (Manassas, VA) were cultured using high-glucose Dulbecco modified Eagle (DMEM) medium (Mediatech Inc., Herndon, VA) supplemented with 10% fetal bovine serum, 5 mM penicillin-streptomycin, and 5 mM L-glutamine. The cells were maintained at 37°C with 5% CO_2_.

### Antibodies

Sug1 antibody was obtained from Novus Biologicals (Littleton, CO). S6a antibody was obtained from Biomol International LP (Plymouth meeting, PA). Mouse IgG control antibody was obtained from Millipore (Lake Placid, NY). RNA Pol II, Ser5p RNA Pol II and Ser2p RNA Pol II and S7 antibodies were obtained from Abcam (Cambridge, MA), CDK9, Hexim and TBP antibodies were obtained from Santa Cruz Biotechnology Inc (Dallas, TX). Horseradish peroxidase (HRP) conjugated mouse antibody was obtained from Promega (Madison, WI), and (HRP)-conjugated rabbit antibody was obtained from Pierce (Rockland, IL). (HRP)-conjugated rabbit IgG veriblot antibody was obtained from Abcam (Cambridge, MA). Anti-Myc and Flag-HRP conjugated antibodies and Anti-mouse HA antibody were obtained from Sigma (St. Louis, MO).

### Plasmids

pBluescript (pBS) S7 and S6a plasmids were generous gifts from Dr. Martin Rochesteiner [39], [40]. These two genes were subcloned into Myc tagged pCMV-3 using the EcoR1 restriction site. Myc-Sug1 was kindly provided by Dr. A. Wani and has been previously described [37]. Flag-Hexim1 plasmid was kindly provided by the Price Lab [41], and HA-CDK9 was kindly provided by the Zhou Lab [42].

### siRNA Constructs and Transfections

Short interfering RNA (siRNA) duplexes were used for transient knockdown of 19S ATPases Sug1, S7, and S6a. The siRNA sequences of Sug1 and S7 siRNA used were 5′-AAGGTACATCCTGAAGGTAAA-3′ and 5′-AACTGCGAGAAGTAGTTGAAA-3′ respectively (Qiagen, Valencia, CA) [7], [37]. The S6a siRNA sequence was a predesigned siRNA directed against PSMC3 [35]. A scrambled siRNA sequence was used as a negative control (Qiagen, Valencia, CA). HeLa cells were transfected with scrambled sequence control siRNA or with ATPase-specific siRNA using HiPerfect transfection reagent (Qiagen) according to the manufacturer’s instructions and were then treated with IFN-γ (500 U/ml) as indicated. Cells were lysed in Nonidet P-40 lysis buffer (NP-40∶1 M Tris pH 8.0, 1 M KCl, 10% NP-40, 0.5 M EDTA, 5 M NaCl, 1 M DTT, dH_2_O), supplemented with complete EDTA-free protease inhibitors (Roche, Florence, SC), and knockdown specificity and efficiency was assessed by Western blotting using anti-Sug1, S7, or S6a primary antibodies at 1∶2000 concentration overnight at 4°C. Mouse-HRP conjugated secondary antibody was used at concentration of 1∶20,000 for 1 h at room temperature.

### RNA Expression

HeLa cells were plated at a density of 8×10^5^ in 10 cm tissue culture plates and 24 h later were transfected with control or ATPase specific siRNA. Forty-eight hours following siRNA transfection, cells were stimulated with IFN-γ (500 U/ml) for 18 h. Six hours prior to harvest, cells were treated with 10 µM of the proteasome inhibitor MG132 (EMD Biosciences) or with 10 µM of the proteasome inhibitor Lactacystin from Biomol International LP (Plymouth meeting, PA). Cells were harvested, washed with cold PBS, centrifuged at 3000 rpm at 4°C for 5 minutes, and total RNA was prepared with 1 ml of Trizol reagent (Invitrogen, Carlsbad, CA) in accordance with the manufacturer’s instructions. The Omniscript Reverse Transcription Kit (Qiagen) was used to generate cDNA from extracted RNA. Gene specific antisense primers (Sigma, Saint Louis, MO) were used for reverse transcription. PCR conditions for all Q-PCR reactions included an initial 10 minute incubation step at 65°C followed by a 60 minute incubation step at 37°C in accordance with the manufacturer’s instructions (Qiagen). The cDNA for CIITA mRNA short (exon IV), CIITA mRNA long (exon VII), and for GAPDH mRNA was amplified using the following reverse primers: 5′-GCTCCAGGTAGCCACCTTCT-3′; 5′-AGCAGTCGCTCACTGGTCTCA-3′; 5′-TAGACGGCAGGTCAGGTCCA-3′. Real-time PCR reactions were carried out on an ABI Prism 7900 (Applied Biosystems, Foster City, CA). Probes for CIITA promoter IV (CIITApIV) and CIITA exon IV and exon VII were 5′ labeled with 6-carboxyfluorescein (FAM) reporter dye and 3′ with N,N,N,N-tetramethyl-6-carboxyrhodamine (TAMRA) quencher dye. Isolated DNA was analyzed by real-time PCR using primers and probes spanning:

- CIITApIV mRNA short(exon IV)–sense sequence 5′-GGGAGAGGCCACCAGCAG-3′, antisense sequence 5′-GCTCCAGGTAGCCACCTTCT-3′, probe sequence 5′-FAM-CTGTGAGCTGCCGCTGTTCCC-3′TAM.
- CIITApIV mRNA long(exonVII)–sense sequence 5′-AACACAGCCCACTTCCTCACA-3′, antisense sequence, 5′-AGCAGTCGCTCACTGGTCTCA-3′, probe sequence 5′FAM-ACTGTGGTGACTGGCAG-3′TAM)
- GAPDH mRNA – sense sequence 5′-GGAAGCTCACTGGCATGGC-3′, antisense sequence 5′-TAGACGGCAGGTCAGGTCCA-3′, probe sequence 5′-FAM-CCCCACTGCCAACGTGTCAGTG-3′TAM)
- 18S rRNA mRNA – sense sequence 5′-GCTGCTGGCACCAGACTT-3′, antisense sequence 5′-CGGCTACCACATCCAAGG-3′, probe sequence 5′-FAM-CAAATTACCCACTCCCGACCCG-3′TAM

Values from real-time PCR reactions were calculated and graphed based on standard curves generated and were normalized to GAPDH message levels. Samples were run in triplicate reactions and were analyzed using the SDS 2.0 program (Applied Biosystems, Foster City, CA). Graphed values represent the percentage difference in the mRNA molecules with respect to ATPase specific siRNA treated and non-treated cells. The highest value is considered 100% and other values were graphed in terms of percentage.

### RNA Expression with ATPase Knockdowns

HeLa cells were plated at a density of 8×10^5^ in 10 cm tissue culture plates and 24 h later were transfected with Sug1, S7, or S6a siRNA. Forty-eight hours following siRNA transfection, cells were stimulated with IFN-γ (500 U/ml) for 18 h. Six hours before harvest, cells were treated with 10 µM MG132 or with 10 µM Lactacystin proteasome inhibitor. Cells were harvested and 10% of the cells were lysed with 1% Nonidet P-40 buffer with protease inhibitors and were analyzed by Western blotting for ATPase knockdown efficiency. The remaining fraction of cell volume was subjected to RNA extraction as above.

### Heat Shock Assay with S6a ATPase Knockdown

HeLa cells were plated at a density of 65,000 cells in a 48 well plate, after 24 hrs the cells were transfected with the indicated plasmids Heat Shock Element promoter tagged with Luciferase (HSE) and with either control siRNA or S6a siRNA using Attractene (Cignal heat shock reporter assay kit, Qiagen, Valencia, CA) according to manufacturer’s instructions. Cells were treated with proteasome inhibitor (MG132) 4 hrs prior to harvest. After 48 hrs, the cells were harvested, washed with cold PBS, and lysed using 1× cell lysis reagent (Promega, Madison, WI). The lysed cell suspension was centrifuged for 2 minutes at 12,000 rpm (Thermo electron 851, Thermo INC, Needham Heights, MA) at 4°C and a Luciferase assay (Promega) was performed according to the manufacturer’s instructions. Ten percent of the cell lysates were normalized for protein concentration by Bradford assay (Bio-Rad), separated by SDS-PAGE, transferred to nitrocellulose and immunoblotted with monoclonal antibody to S6a and secondary goat anti-mouse horseradish peroxidase conjugated antibody. The negative control is a mixture of non-inducible reporter construct and constitutively expressing Renilla luciferase construct provided in the kit. Positive control is an inducible transcription factor-responsive construct expressing firefly luciferase, and a constitutively expressing Renilla luciferase construct; both are provided in the kit.

### Co-immunoprecipitation

For over expression co-immunoprecipitation experiments, HeLa cells were plated at a density of 8×10^5^ in 10 cm tissue culture plates and transfected with 5 µg of Myc-Sug1, Myc-S7, or Myc-S6a and 5 µg of pcDNA, HA-CDK9, or Flag-Hexim plasmids using Fugene 6 (Roche, Indianapolis, IN) according to manufacturer’s instruction. After 24 h, cells were harvested and lysed using RIPA lysis buffer (1 M Tris pH 8.0, 5 M NaCl, 10% NP-40, 5% DOC, 10% SDS, 1 M DTT, dH_2_O) supplemented with complete EDTA-free protease inhibitor (Roche, Indianapolis, IN). Cell lysates were centrifuged, normalized for protein concentration, and pre-cleared with 50 µl mouse IgG (Sigma, Saint Louis, MO) and were immunoprecipitated overnight with 10 µg antibody against Sug1, S7, or S6a. Negative control samples were immunoprecipitated overnight with 10 µg of mouse IgG. Immune complexes were isolated with Myc beads (for 19S ATPases) (Sigma), Flag beads (for Flag-Hexim 1) (Sigma) or HA beads (for HA-CDK9) (Sigma) on a rotator at 4°C and complexes were denatured with Leammli buffer (Bio-Rad), boiled, and separated by SDS-PAGE gel electrophoresis. Gels were transferred to nitrocellulose and co-immunoprecipitated complexes were detected by immunoblotting using mouse anti-Myc-HRP to detect Myc-tagged ATPases, mouse anti-Flag-HRP to detect Flag-Hexim 1, and mouse anti-HA antibody to detect HA-CDK9. HRP conjugates were detected with the HyGlo Chemiluminiscent reagent kit (Denville). Equal loading was determined in non-immunoprecipitated lysates by immunoblotting of total protein.

For endogenous co-immunoprecipitation experiments, 7×10^6^ HeLa cells were lysed in NP-40 lysis buffer with protease inhibitors for 30 minutes on ice. Lysates were centrifuged, normalized for protein concentration, precleared with 50 µl mouse IgG (Sigma-Aldrich) and were immunoprecipitated with 5 µg of antibody against Sug1, S7, or S6a respectively. Positive control samples were immunoprecipitated with 5 µg of Hexim, CDK9 or Ser5p RNA pol II antibody and negative control samples were immunoprecipitated with 5 µg of mouse IgG. Isolated immune complexes were denatured with Leammli buffer, boiled and separated by SDS-PAGE gel electrophoresis. Gels were transferred to nitrocellulose and were individually immunoblotted for endogenous Hexim, CDK9 or Ser5p RNA pol II. Equal loading was determined in non-immunoprecipitated lysates by immunoblot of total protein.

### Chromatin Immunoprecipitation (ChIP)

HeLa cells were plated at a density of 2.5×10^6^ in 15 cm-tissue culture plates and were treated with IFN-γ (500 U/ml) for 0.5, 2, 3, 4, or 18 hrs. Following IFN-γ stimulation, cells were crosslinked with 1% formaldehyde for 8 minutes at room temperature; crosslinking was stopped by the addition of 0.125 M glycine for 5 minutes at room temperature. Cell nuclei were isolated and concentrated by lysing in cell lysis buffer (5 mM PIPES pH 8, 85 mM KCl, 1% igepal) and protease inhibitors for 15 minutes on ice. The cell lysate was centrifuged at 2100 rpm for 5 minutes at 4°C. The supernatant was discarded and the pellet was resuspended in SDS lysis buffer (1% SDS, 10 mM EDTA, 50 mM Tris pH 8.0, dH_2_O) and protease inhibitors for 25 minutes on ice followed by flash freezing in liquid nitrogen. Lysed nuclei were sonicated using a Bioruptor water bath sonicator for 30 sec “On” and 30 sec “Off” 3 times to generate an average of 500 bp of sheared DNA. The sonicated lysates were pre-cleared with salmon-sperm coated agarose beads (Upstate) and lysates were divided equally. One half of the lysate was immunoprecipitated with 5 µg of antibody to Sug1, S7, S6a, or RNA Pol II overnight at 4°C. The other half of the lysate was immunoprecipitated with control antibody. Immunoprecipitated proteins were isolated during 2 h incubation with 60 µl of salmon-sperm coated agarose beads. Immunoprecipitated samples were washed for 3 minutes at 4°C with the following buffers: low salt buffer (0.1% SDS, 1% Triton X-100, 2 mM EDTA, 20 mM Tris pH 8.0, 150 mM NaCl, dH_2_O), high salt buffer (0.1% SDS, 1% Triton X-100, 2 mM EDTA, 20 mM Tris pH 8.0, 500 mM NaCl, dH_2_O), LiCl buffer (0.25 M LiCl, 1% NP40, 1% DOC, 1 mM EDTA, 10 mM Tris pH 8.0, dH_2_O) and 1X TE buffer; DNA was then eluted with SDS elution buffer (1% SDS, 0.1 M NaHCO_3_, dH_2_O). After DNA elution, crosslinks were reversed overnight with 5 M NaCl at 65°C followed by treatment with proteinase K for 1 hr at 45°C and immunoprecipitated DNA was isolated using a phenol:chloroform:isopropanol mix (Invitrogen) as per the manufacturer’s instructions. Real-time PCR reactions were carried out on an ABI prism 7900 (Applied Biosystems, Foster City, CA). CIITA promoter IV, CIITA exon IV, and CIITA exon VII were labeled 5′ with FAM reporter dye and 3′ with TAMRA quencher dye. Isolated DNA was analyzed by real-time PCR using primers spanning:

- CIITApIV promoter (Sense sequence, 5′-CAGTTGGGATGCCACTTCTGA-3′; Antisense sequence, 5′-TGGAGCAACCAAGCACCTACT-3′; Probe sequence, 5′-6 FAM-AAGCACGTGGTGGCC-3′TAM),
- CIITApIV exon IV(Sense sequence 5′-TGCCCTAATACCTGACGACCAT-3′, Antisense sequence 5′-AAGCCCAAGGTGAGTCTCTATTGT-3′, Probe sequence 5′-6 FAM-CAGTCAGACCCCTCTCCCCAAGGTG-3′TAM),
- CIITApIV exon VII region (Sense sequence 5′-AACACAGCCCACTTCCTCACA-3′, Antisense sequence 5′-AGCAGTCGCTCACTGGTCTCA-3′, Probe sequence 5′-6 FAM-ACTGTGGTGACTGGCAG-3′ TAM)
- CD4 (Sense sequence, 5′-CACAGGAATGTGCTCTGC-3′, Antisense sequence 5′-CAGTCTCTGACCTCTGGAAG-3′, Probe sequence 5′-6FAM-ACAGCTCTGGCCACCTTCTCTTGCA-3′ TAM)

Values from real-time PCR reactions were calculated and graphed based on standard curves generated, were run in triplicate reactions, and were analyzed using the SDS 2.0 program.

### Chromatin Immunoprecipitation with ATPase Knockdown

HeLa cells were plated at a density of 2.5×10^6^ in 10-cm tissue culture plates and were transfected with control siRNA or with ATPase-specific siRNA (Qiagen). Cells were stimulated with IFN-γ (500 U/ml) as indicated and 10% of the total cell volume was lysed with 1% Nonidet P-40 buffer with protease inhibitors and cell lysates were analyzed by Western blotting for ATPase knockdown specificity and efficiency. The remaining fraction of cell volume was subjected to ChIP assay as described above.

## Results

### The Proteasomal 19S ATPases Sug1, S7, and S6a are Recruited to the CIITApIV Proximal Promoter

**Figure 2:**
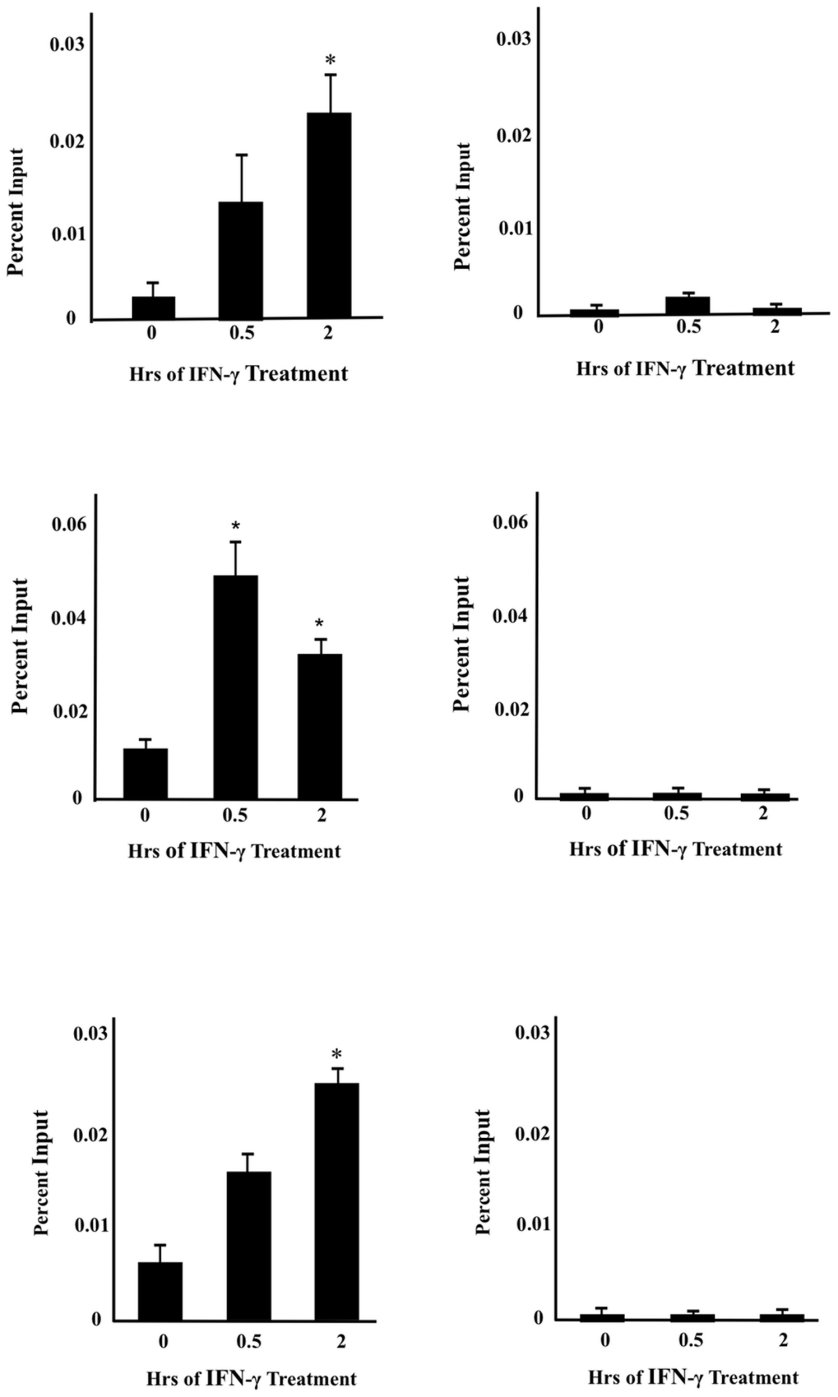
19S ATPases associate with the CIITA pIV proximal promoter. (A, C,E) ChIP assays were carried out in HeLa cells stimulated with IFN-γ for 0–2 hrs. Cell lysates were immunoprecipitated (IP’d) with control antibody or with antibody to endogenous 19S ATPase S6a, Sug1, or S7 and associated DNA was isolated and analyzed by real-time PCR using primers and probe spanning the CIITApIV proximal promoter. Real time PCR values were normalized to the total amount of DNA in the reaction (Input). IP values are represented as ATPase binding to CIITApIV promoter DNA relative to unstimulated samples. (B,D,F) ChIP signal at the inactive gene CD4. The control IgG values were 0.004±0.001. Values for control IgG and either Sug1 IP, S7 IP or S6a IP represent the mean ± SEM of three biologically independent experiments * p<0.05.

We have previously shown that Sug1 associates with the MHC II promoter, regulates recruitment of CIITA and histone modifying enzymes to the promoter, and subsequently plays important roles in MHC II gene expression [7]. It has previously been demonstrated that yeast Rpt6 (Human Sug1) is required for efficient transcription elongation of RNA Pol II [43]. To analyze the role of the mammalian 19S ATPases in transcription elongation of additional genes, we evaluated whether Sug1 and other 19S ATPases influence transcription of inducible CIITApIV. ChIP assays were performed to determine whether ATPases Sug1, S7, or S6a directly bind to CIITApIV proximal promoters. HeLa cells were stimulated with IFN-γ, immunoprecipitated with antibodies to endogenous Sug1, S7, or S6a, and analyzed by real-time PCR with primers and probes spanning the CIITApIV promoter. Sug1, S7, and S6a associate with the CIITApIV promoter within 30 minutes of IFN-γ stimulation and their binding is significant following two hours of stimulation (Figure 2A, C, E). These results indicate Sug1, S7, and S6a are inducibly recruited to the CIITApIV promoter.

### CIITA Long Transcripts are Significantly Decreased in the Absence of 19S ATPases

**Figure 3:**
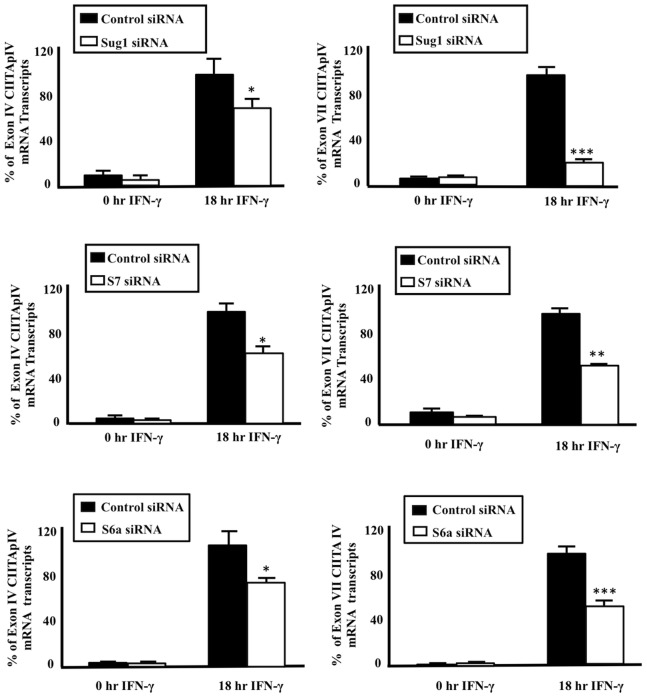
Reduced expression of 19S ATPases via siRNA negatively impacts the generation of long transcripts from CIITA pIV. (A–B, D–E, G–H) Cells were transfected with siRNA, and mRNA was quantitated using CIITA mRNA primers and probes specific for transcripts from CIITA exon IV and exon VII. CIITA mRNA generated was normalized to GAPDH. Data shown represent the mean ± SEM of three biologically independent experiments. (C, F, I) Expression of Sug1, S7, and S6a was specifically decreased using ATPase specific siRNA (Figure S1A, S1B, and S1C). Blots shown are representative of three biologically independent experiments.

The 19S regulatory subunit of the 26S proteasome consists of six paired ATPases (Sug1 and S6b; S7 and S4; and S6a and S10b), which recognize, unfold, and direct polyubiquitinated proteins towards the 20S proteolytic core for degradation. We have previously shown that 19S ATPases (Sug1, S7, and S6a) co-immunoprecipitate with CIITA, regulate the binding of CIITA to the MHC II promoter, and promote transcription initiation of MHC-II genes [7]. To further understand the functions of these ATPases in the transcription of CIITApIV genes, CIITApIV mRNA was extracted from HeLa (human epithelial) cells which had been transfected with Sug1, S6a, or S7 siRNA duplexes in the presence and absence of IFN-γ stimulation. cDNA was prepared from the extracted mRNA using reverse primers specific for the CIITApIV exons IV and VII which correlate to short and long transcripts respectively; mRNA yields from specific samples were then quantified using real-time PCR. In cells transfected with Sug1 siRNA, the generation of both CIITApIV mRNA short and long transcripts is significantly reduced as compared to control siRNA treated cells (Figure 3A & 3B). Similarly, cells transfected with either S7 or S6a siRNA exhibit a reduced generation of short and long transcripts. In each instance the generation of CIITApIV long transcripts is significantly more impacted than is the generation of short transcripts indicating the impact of ATPase deficiency increases as transcription proceeds (compare Figure 3A & 3B; 3C & 3D; 3E & 3F). siRNA knockdown efficiency of the ATPases is shown in Figure S1A, S1B, and S1C.

### Effect of Proteasome Inhibition on CIITApIV Transcription

**Figure 4:**
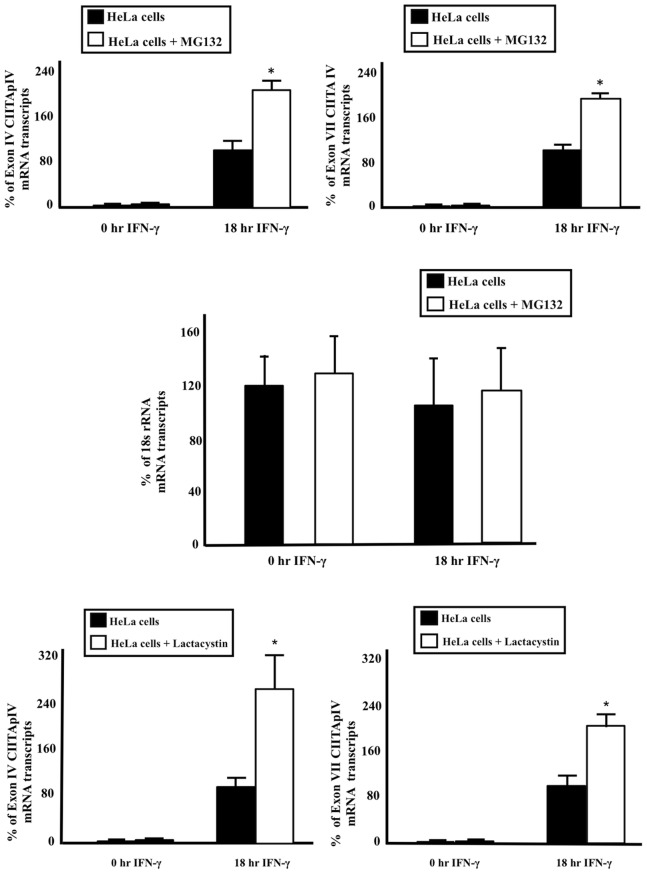
Effects of 19S ATPase knockdown on CIITApIV transcription are independent of effects on degradation. (A–B, D–E) HeLa cells were stimulated with IFN-γ as indicated and were harvested four hrs post treatment with 10 µM MG132 or 10 µM Lactacystin. mRNA was extracted and cDNA was generated using indicated reverse primers followed by amplification via real-time PCR. CIITA mRNA transcripts were obtained using primers and probes specific for CIITA exon IV and exon VII were normalized to GAPDH. (C) 18S rRNA transcripts for control and MG132 treated cells were obtained using primers and probe specific for 18S rRNA and were normalized to GAPDH. The 18 hr control sample was set to 100%. Data shown represents the mean ± SEM of three biologically independent experiments.

We previously demonstrated treatment of cells with S6a siRNA and Sug1 siRNA moderately reduces 26S-mediated proteolysis [35], [37]. In the present work, the 26S proteasome inhibitors MG132 and Lactacystin were used to determine whether the negative effect of 19S ATPase knockdown on CIITApIV transcription is a result of inhibition of the proteolytic function of the proteasome. HeLa cells were stimulated with IFN-γ and were treated with MG132 (10 µM) 4 hrs prior to harvesting. CIITA mRNA was extracted, and cDNA was generated using CIITApIV Exon IV, VII and 18S rRNA reverse primers; mRNA yields for exons IV, VII and 18S rRNA were then quantified by real-time PCR. Treatment of cells with MG132 increases the number of both short (Figure 4A) and long (Figure 4B) CIITA mRNA transcripts, but there was no significant change in 18S rRNA transcripts in control and MG132 treated samples (Figure 4C). Similarly, Lactacystin (10 µM) treatment of HeLa cells shows a similar increase in CIITA mRNA transcript levels (Figure 4D & 4E). These results indicate the role of 19S ATPases in the generation of CIITApIV mRNA transcripts is independent of the proteolytic function of the 26S proteasome.

### S6a siRNA does not Activate Heat Shock Response

**Figure 5:**
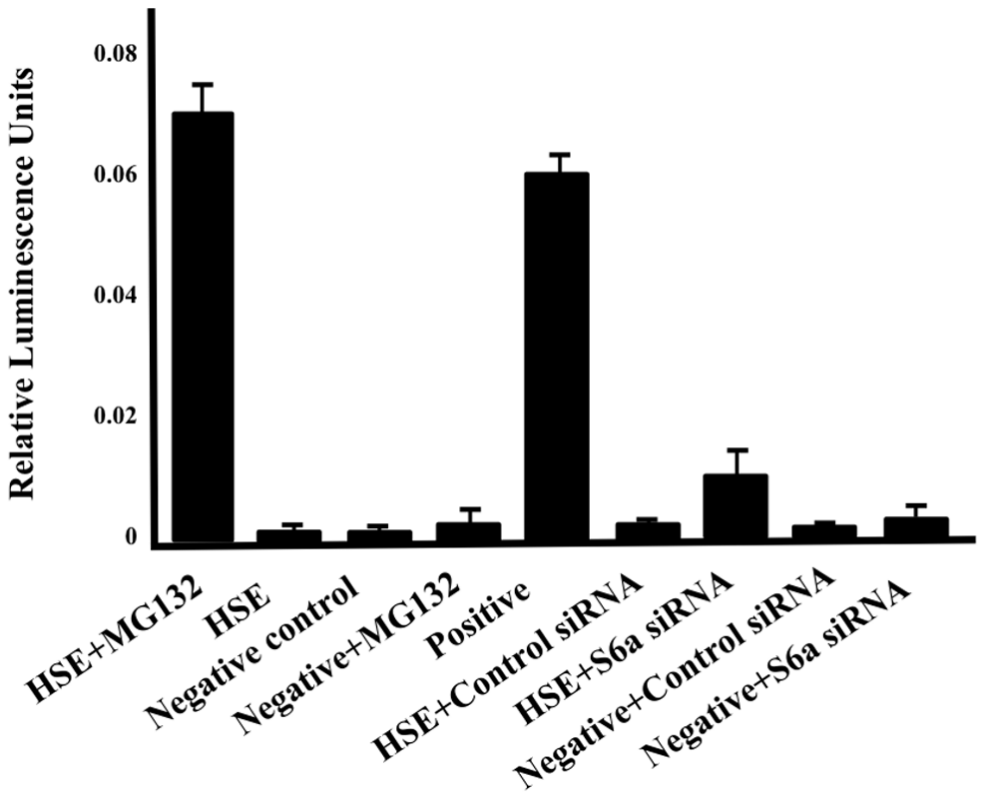
Knockdown of 19S ATPases does not activate the heat shock response. HeLa cells were transfected with HSE-Luciferase reporter, control siRNA, or S6a siRNA and were treated with MG132 six hrs prior to harvest. Cells were harvested following 48 hrs of incubation, lysed in cell lysis buffer, and analyzed by Luciferase assay. Luciferase readings obtained were normalized by Bradford assay. Data shown represents values obtained from three independent experiments. The negative control is a mixture of non-inducible reporter construct and constitutively expressing Renilla luciferase construct. The positive control is an inducible transcription factor-responsive construct expressing firefly luciferase, and a constitutively expressing Renilla luciferase construct.

Knockdown of 19S ATPases could also indirectly affect transcription by altering steady state protein levels and activating a heat shock response [44]. To determine if 19S ATPase siRNA activates the heat shock response, HeLa cells were transfected with the Heat Shock Element (HSE) promoter tagged with Luciferase and with either control siRNA or S6a siRNA, or were treated with proteasome inhibitor MG132. Cells were harvested after 48 hrs incubation. The results shown in Figure 5 indicate a robust heat shock response in cells treated with MG132 and in cells transfected with the positive control, but no significant heat shock response in cells treated with S6a siRNA. Together these findings indicate that the effect of 19S ATPase siRNA in mediating reductions in CIITApIV mRNA transcripts is due to non-proteolytic roles of the 19S ATPases.

### The 19S ATPases Sug1, S7, S6a Bind within the Coding Region of CIITApIV Gene

**Figure 6:**
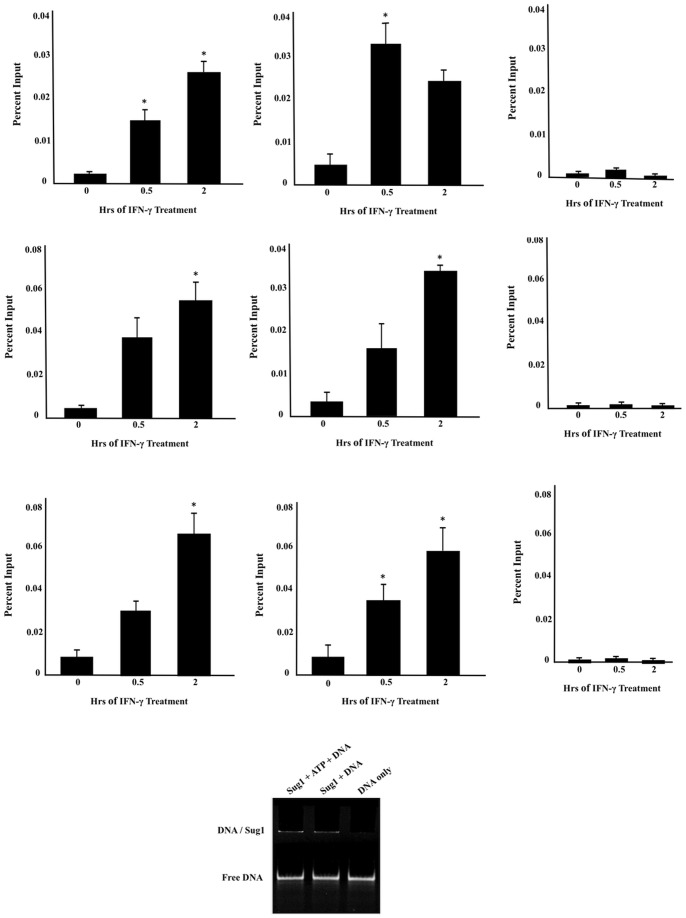
19S ATPases bind CIITA pIV within the coding region. (A–I) ChIP assays were carried out in HeLa cells stimulated with IFN-γ for 0–2 hrs. Cell lysates were IP’d with control antibody or with antibody to endogenous Sug1 (A and B), S7 (D and E), or S6a (G and H) and associated DNA was isolated and analyzed by real-time PCR using primers and probes spanning CIITApIV exon IV (A, C, E) and exon VII (B, D, F). Real time PCR IP values were normalized to the total amount of DNA (input); IP values are represented as ATPase binding to CIITApIV exon IV or exon VII DNA relative to unstimulated samples. (C,F,I) ChIP signal at the inactive gene CD4. The control IgG values were 0.005±0.001. Values for control and IP represent mean ± SEM of three biologically independent experiments. *p<0.05, **p<0.005. G. Mobility shift assay of Sug1 with a 90 nucleotide single stranded DNA on a native 8% polyacrylamide gel with a tris-borate magnesium running buffer; 0.7****µM DNA, 0.85 µM sug1, and 500 µM ATP. DNA was visualized with SYBER Green II dye.

To evaluate whether 19S ATPases move from promoters into actively transcribing mammalian genes, we investigated the binding of 19S ATPases to CIITApIV coding sequences using ChIP assays. We designed primers and probe sets spanning exons IV and VII of CIITApIV and performed ChIP assays in HeLa cells for Sug1, S7 and S6a. Cells were stimulated with IFN-γ (0–2 hrs) as indicated and crosslinked and sonicated lysates were subjected to immunoprecipitation (IP) with antibodies against endogenous Sug1, S7, or S6a. Following IP, associated DNA was isolated and analyzed by realtime PCR using primers spanning CIITA exon IV and exon VII. Binding of Sug1 to CIITApIV exons IV and VII was observed over a time-course of IFN-γ stimulation with significant binding at 2 hr and 0.5 hr respectively (Figure 6A & B). Similarly, 19S ATPases S7 and S6a bind to CIITApIV exon IV and exon VII over IFN-γ stimulation with significant binding at 2 hr (Figure 6D, E & 6G, H). To probe for potential direct interactions between the 19S ATPases and DNA, gel mobility shift assays were conducted in which purified Myc tagged Sug1 was added to a 90 nucleotide single-stranded DNA oligonucleotide. As shown in Fig. 6J, lanes in which Sug1 is added to single stranded DNA exhibit a precipitant in the wells indicating an interaction with Sug1. Of note, similar assays with small double-stranded DNA oligonucleotides did not exhibit a similar precipitation (data not shown). Together these data indicate that the 19S ATPases bind to CIITA coding regions, supporting potential roles in RNA Pol II processivity.

### The 19S ATPases Sug1, S7, S6a Associate with Components of the Elongation Factor PTEFb Complex (Hexim and CDK9)

**Figure 7:**
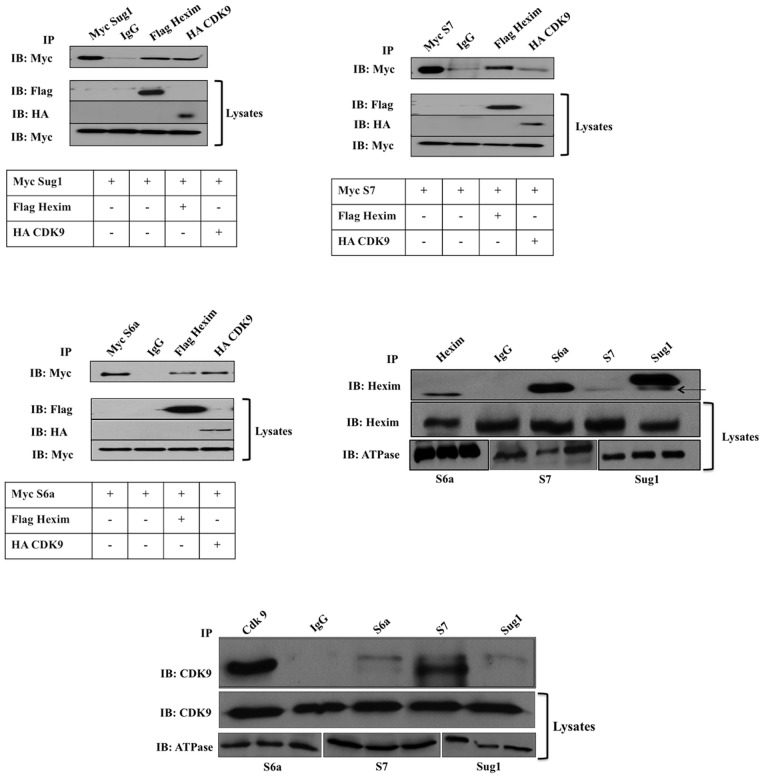
19S ATPases associate with elongation factors Hexim and CDK9. (A–C) HeLa cells were co-transfected with Myc tagged S6a, S7, or Sug1 and Flag tagged Hexim or HA tagged CDK9 as indicated. Cells were lysed and IP’d with Myc beads (lane 1) as a positive control, mouse isotype IgG (lane 2) as a negative control, flag beads (lane 3), and HA beads (lane 4). IP samples (top panel) and lysates (bottom panel) were IB’d for Myc, Flag, and HA as indicated. (D–E) HeLa cells were lysed and IP’d with either Hexim or CDK9 (lane 1) as a positive control, mouse isotype IgG (lane 2) as a negative control, or with S6a (lane 3), S7 (lane 4), and Sug1 (lane 5). IP samples (top panel) and lysates (bottom panel) were IB’d for Hexim or CDK9 as indicated. Results shown are indicative of data from three biologically independent experiments.

It was previously reported that the 19S ATPases associate with transcription factors and promote active transcription in yeast [43]. As the 19S ATPases bind to the CIITApIV proximal promoter, co-immunoprecipitation (co-IP) assays were used to determine whether the 19S ATPases also associate with transcription factors Hexim and CDK9 which are required for productive elongation. Protein-protein interactions were initially determined using over expression co-IP assays in which HeLa cells were transfected with Myc-tagged ATPases, with Flag-Hexim or HA-CDK9 and, following IP for either Flag-Hexim or HA-CDK9, were then immunoblotted (IB) for Myc. The IP blot shown in Figure 7A indicates association of Hexim (lane 3) and CDK9 (lane 4) with Sug1. Similarly, the IP blots shown in Fig. 7B & 7C indicate the association of S7 and S6a ATPases with Hexim (lanes 3) and CDK9 (lanes 4). The efficiency of transfections and equal loading of cell lysates was confirmed by IB of Myc, HA, and Flag- tagged proteins. Co-immunoprecipitation experiments were next performed with endogenous proteins to determine if endogenous 19S ATPases (S6a, S7, Sug1) interact with endogenous Hexim and CDK9. Each of the endogenous 19S ATPases associate with Hexim (Figure 7D) while only endogenous S7 demonstrated pronounced interaction with CDK9 (Figure 7E). Together these results demonstrate the 19S ATPases are in complexes with the Hexim1 and CDK9 components of PTEFb complex, which in turn regulates transcription elongation of CIITA genes.

### The 19S ATPases Associate with Ser5 Phosphorylated-RNA pol II

**Figure 8:**
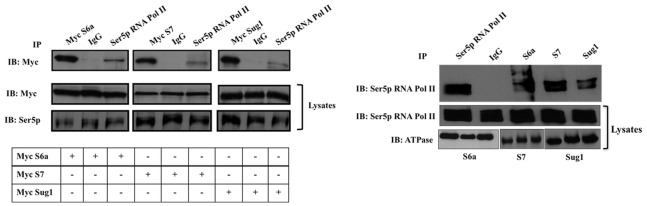
19S ATPases associate with Ser5 phosphorylated RNA pol II (Ser5p-RNA pol II). (A) HeLa cells were transfected with myc tagged S6a, S7 or Sug1 as indicated. Cells were lysed and IP’d with myc beads (first lane, top panels) as a positive control, with mouse isotype IgG (second lane, top panels) as a negative control, and with Ser5p-RNA Pol II antibody (third lane, top panels). IP’d samples (top panels) and lysates (middle and bottom panels) were IB for myc ATPases or for Ser5p-RNA pol II as indicated. (B) HeLa cells were lysed and IP’d with Ser5p-RNA Pol II (lane 1) as a positive control, mouse isotype IgG (lane 2) as a negative control, or with S6a (lane 3), S7 (lane 4), or Sug1 (lane 5). IP samples (top panel) and lysates (bottom panel) were IB’d Ser5p-RNA Pol II as indicated. Results shown are indicative of data from three biologically independent experiments.

Transcription in eukaryotes is initiated by the recruitment of RNA Pol II to promoter regions. RNA Pol II initiates synthesis of mRNA upon association with basal transcription factors and phosphorylation of its C terminal domain (CTD). Once preinitiation complexes form, RNA Pol II is phosphorylated on Serine 5 (Ser5p) by CDK7 of TFIIH and mRNA synthesis begins [45]. A co-IP assay was performed to determine if 19S ATPases associate with Ser5p-RNA Pol II. HeLa cells were transfected with Myc-tagged ATPases, IP’d with Ser5p-RNA Pol II antibody, and IB’d for Myc. The IP blot shown in Figure 8 indicates association of expressed S6a, S7 and Sug1 ATPases with Ser5p-RNA Pol II (lanes 3, 6, 9). The efficiency of transfections and equal loading of cell lysates was confirmed by IB of Myc tagged proteins and of Ser5p-RNA Pol II. Analysis of co-IP of endogenous proteins also indicate association of each of the 19S ATPases with Ser5p-RNA Pol II (Figure 8B). Additional co-IP experiments with Ser2p-RNA pol II indicated only weak interactions (faint bands) between expressed S6a, S7, and Sug1 ATPases and Ser2p-RNA pol II (data not shown). Analysis of co-IP of endogenous proteins indicates strong association of 19S ATPases with Ser5p-RNA Pol II (Figure 8B).

### Knockdown of 19S ATPases Decreases RNA Pol II Phosphorylation but does not Impact Degradation

**Figure 9:**
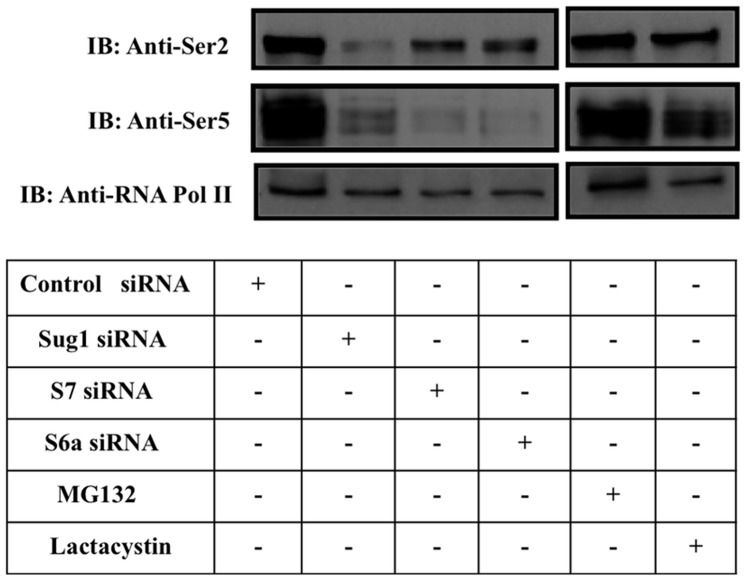
Reduced expression of 19S ATPases decreases phosphorylated forms of RNA Pol II. A. HeLa cells were transfected with siRNA or were treated with proteasome inhibitors as indicated. Cells were harvested following 48hrs of siRNA incubation. Cell lysates were IB’d with Ser2p-RNA pol II antibody (top panels), Ser5p-RNA pol II (middle panels), or with RNA pol II antibody (bottom panels). Cells treated with proteasome inhibitors serve as a positive control for degradation dependent effects. Results shown are indicative of data from three biologically independent experiments. Sug1, S7, and S6a protein expression was effectively decreased using specific siRNA. Actin blots demonstrate loading and siRNA specificity controls (Figure S2A and S2B).

Eukaryotic RNA Pol II consists of a C terminal domain composed of a series of heptad repeats [46]. The phosphorylation pattern of the CTD changes during transcription and serves as a flexible scaffold for binding nuclear factors required for specific transcription stages [45], [47]. To understand if 19S ATPases affect the regulation of RNA Pol II phosphorylation, we next determined the effect of 19S ATPase knockdown on levels of non-phosphorylated RNA pol II and on levels of phosphorylated RNA pol II (Ser2p-RNA Pol II and Ser5p-RNA Pol II). HeLa cells were transfected with control siRNA or with ATPase specific siRNA and 48 hrs later cell lysates were IB’d using RNA Pol II, Ser2p-RNA Pol II, or Ser5p-RNA Pol II antibodies. The IB shown in Figure 9 (bottom panel) indicates there is no change in non-phosphorylated RNA pol II levels in 19S ATPase specific siRNA treated samples versus control siRNA treated samples and MB132 treated samples. In comparison, there is a decrease in levels of Ser5p-RNA Pol II and, to a lesser extent Ser2p-RNA Pol II (top and middle panels), in 19S ATPase siRNA treated samples versus control siRNA treated samples and MG132 treated samples. The knockdown efficiency and specificity of the 19S ATPase specific siRNA constructs is shown in Figure S2A and S2B.

### Knockdown of 19S ATPases Sug1, S7, or S6a Decreases RNA Pol II and TBP Recruitment to CIITApIV Proximal Promoter

**Figure 10:**
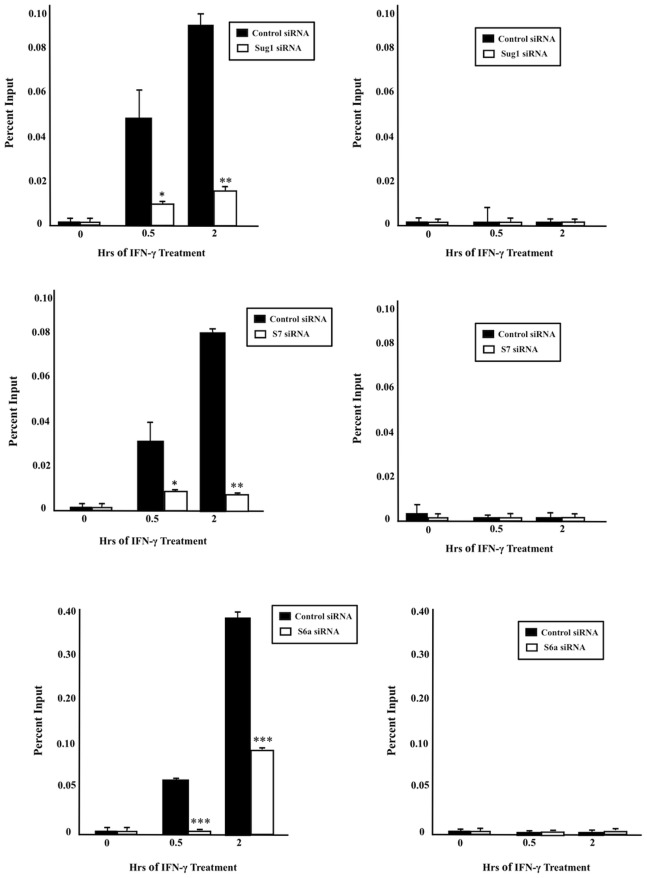
Reduced expression of 19S ATPases decreases RNA pol II recruitment to the CIITApIV proximal promoter. (A,C,E) ChIP assays were carried out in HeLa cells transfected with ATPase specific or with control siRNA and stimulated with IFN-γ for 0–2 hrs. Cell lysates were crosslinked, sonicated, lysed, and IP’d with either antibody against endogenous RNA pol II or with control antibody (IgG). Associated DNA was analyzed via real-time PCR using primers and probe specific for the CIITApIV proximal promoter. Real time PCR IP values were normalized to total amount of reaction DNA (Input). The values for control IP and RNA Pol II IP represent the mean of three biologically independent experiments *p<0.05, **p<0.005, ***p<0.0005 versus control siRNA. (B, D, F) ChIP signal at the inactive gene CD4. Sug1, S7, and S6a protein expression was effectively decreased using ATPase specific siRNA (Figure S3A, S3B, and S3C).

**Figure 11:**
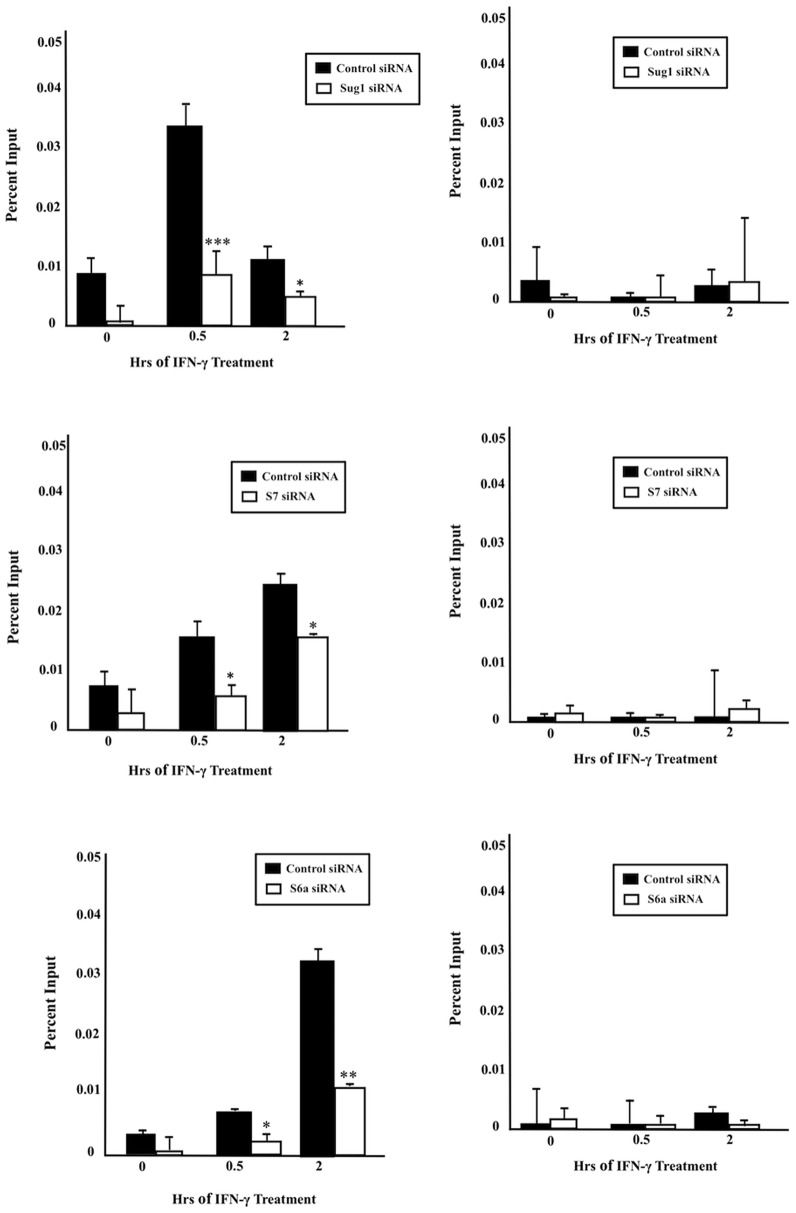
Reduced expression of 19S ATPases decreases TBP recruitment to the CIITApIV proximal promoter. (A, C, E) ChIP assays were carried out in HeLa cells transfected with ATPase specific or with control siRNA and stimulated with IFN-γ for 0–2 hrs. Cell lysates were crosslinked, sonicated, lysed, and IP’d with either antibody against endogenous TBP or with control antibody (IgG). Associated DNA was analyzed via real-time PCR using primers and probe specific for the CIITApIV proximal promoter. Real time PCR IP values were normalized to total amount of reaction DNA (Input). The values for control IP and TBP IP are representative data *p<0.05, **p<0.005, ***p<0.0005 versus control siRNA. (B, D, F) ChIP signal at the inactive gene CD4. Sug1, S7, and S6a protein expression was effectively decreased using ATPase specific siRNA (Figure S4A, S4B, and S4C).

Regulation of protein coding genes is mediated by RNA Pol II and multiple transcription factors at various steps of the transcription process. Transcription initiation is accomplished by the recruitment of RNA Pol II to promoter sites and the formation of a pre-initiation complex with basal transcription factors [48]. To assess whether the 19S ATPases aid in the recruitment of RNA Pol II to CIITApIV proximal promoters, ChIP assays (using antibody specific for RNA Pol II and primers and probe specific for the CIITApIV promoter region) were performed with cells treated with either control siRNA or with ATPase specific siRNA. As shown in Figure 10A, in cells treated with control siRNA, RNA Pol II inducibly binds to the CIITApIV proximal promoter over a time course of IFN-γ stimulation. Cells transfected with Sug1 siRNA exhibit significantly decreased binding of RNA Pol II to the CIITApIV proximal promoter as compared to control siRNA treated samples. A similar trend was observed with S7 and S6a siRNA treated cells (Figure 10B, 10C). Efficiency of the knockdown of ATPase using siRNA is shown in Figure S3A, S3B, and S3C. Among the general transcription factors, TFIID factor is a complex composed of TATA binding protein (TBP) and TBP-associated factors (TAFIIS) and is required for transcription machinery. To assess whether the 19S ATPases aid in the recruitment of TBP to CIITApIV promoter, ChIP assays (using antibody specific for TBP and primers and probe specific for the CIITApIV promoter region) were performed with cells treated with either control siRNA or with ATPase specific siRNA. As shown in Figure 11A, in cells treated with control siRNA, TBP inducibly binds to the CIITApIV promoter over the time course of IFN-γ treatment. Cells transfected with Sug1siRNA exhibit significant decrease in binding of TBP to the CIITApIV promoter as compared to control siRNA treated cells. A similar trend was observed with S7 and S6a siRNA treated cells (Figure 11B, 11C). Efficiency of the knockdown of ATPase using specific siRNA is shown in Figure S4A, S4B and S4C. These studies indicate the 19S ATPases play critical roles in RNA Pol II and TBP recruitment at CIITA genes.

## Discussion

Proteasomal proteins are crucial regulators of transcriptional activities both dependent on, and independent of, protein degradation. While the stage is set for novel developments in our understanding of gene expression, the roles of individual proteasome components in transcription remain to be determined. The 19S ATPases were initially found to associate with actively transcribed genes and to facilitate recruitment of transcription factors to active genes in yeast where 19S ATPases play important, but undefined, roles in RNA Pol II dependent elongation [43]. We have previously demonstrated that binding of 19S ATPases at the mammalian MHCII promoter mediates transcription initiation by stabilizing the binding of activating histone modifying enzymes and transcription factors [7], [35]. We show now that representatives of each of the 19S ATPase heterodimers are inducibly recruited to another mammalian gene, the CIITApIV promoter (Figure 2) and, that upon activation of transcription, 19S ATPases move into CIITApIV exons (Figure 6).

Since chromatin structure was recognized as repeating units of histones and DNA in nucleosome cores, it has been proposed that the function of chromatin is to regulate transcription [49]. Chromatin regulators transiently remodel chromatin in response to cellular signals in order to change the accessibility of DNA to transcription factors and polymerases [50]. As the 19S ATPases bind throughout CIITApIV genes, our initial focus was to determine the impact of the ATPases on the generation of transcripts from CIITApIV. Depletion of the 19S ATPases Sug1, S7, or S6a decreases IFN-γ induced transcription of CIITApIV with significant impact on the generation of longer CIITApIV transcripts (Figure 3). These observations indicate the 19S ATPases are involved in regulating chromatin: in the absence of 19S ATPases, the further the polymerase has to move, the more difficult the journey ahead.

Depleting 19S ATPases might lead to the malfunction of the proteasome and therefore impair degradative processes. To address this concern, we previously demonstrated that degradation continues in cells in which 19S ATPases have been knocked down [37]. These data support recent findings of the existence of cellular pools of 19S ATPases and also support our hypothesis that 19S ATPases have non-proteolytic roles in regulating transcription. We extend these observations here by demonstrating that while 19S ATPase knockdown inhibits CIITApIV transcription, inhibition of proteasome activity significantly increases CIITApIV transcription (Figure 4). These data further indicate the 19S ATPases have functions that are independent of proteasome activity but which are essential in the regulation of transcription. Our current findings also support our previous observations that unlike ATPase knockdown, proteasome inhibition does not affect the regulation of acetylation and methylation of histones H3 and H4 [7], [35]–[38].

Finally, very efficient knockdown of 19S ATPases could lead to the accumulation of mis-folded and partially degraded proteins, and the activation of a heat shock response, similar to that caused by temperature induced protein mis-folding [51]. We observe here however that siRNA mediated knockdown of the 19S ATPases does not activate a heat shock response (Figure 5). Together these data also indicate the effects of 19S ATPase knockdown on transcription of CIITApIV are direct, are due to nondegradative roles for 19S ATPases, and are not the result of dysregulated proteolysis.

The expression of protein coding genes is carried out by RNA Pol II and various transcription factors [52] and is controlled at multiple levels [53], [54]. RNA Pol II escape from promoter regions and the transition of RNA Pol II to an elongating complex is highly regulated and specific phosphorylation events of the RNA Pol II CTD (C-terminal domain) are required for transcription initiation and elongation [55], [56]. RNA Pol II Ser 5 phosphorylation is required for the transition from initiation to elongation whereas Ser 2 phosphorylation is required for elongation [56]. These phosphorylation events are mediated by kinase TFIIH (CDK7), the mediator complex, and the kinase PTEF-b (CDK9). As the 19S ATPases are required for transcription from CIITApIV, we next determined if these ATPases associate with transcription elongation factors and with the phosphorylated forms of RNA Pol II.

We first showed that the 19S ATPase Sug1 interacts with DNA (Figure 6G) and that both tagged and endogenous proteins (ATPases Sug1, S7, and S6a) interact with components of PTEFb and with Ser5p-RNA Pol II (Figure 7 & 8). Variation in the intensity of interactions seen in co-IP experiments is evidence of the independent functions of 19S ATPases in regulating transcription [57]. PTEF-b exists in two forms, active and inactive, where the inactive form of PTEF-b is bound to Hexim1 and 7SK RNA. Upon cytokine stimulation, PTEF-b is dissociated from the inactive complex bound by Hexim-1 and 7SK RNA and is recruited to actively transcribing genes [37]–[39]. Association of 19S ATPases with factors and complexes associated with transcription elongation was not surprising as the ATPases were found distributed throughout CIITApIV exons. It is noteworthy that Ser2p of RNA Pol II is required for maintaining both global and gene associated levels of H2B monoubiquitination as previous work by Ezhkova and Tansey has shown that H2B K123 ubiquitination is required to recruit 19S ATPases [58]. There is also evidence indicating that the 19S regulatory particle base is required for SAGA recruitment and that particularly the 19S ATPase Sug1 interacts with the SAGA complex and aids in transcription [59]. Together, these data suggest the 19S ATPases are recruited to promote chromatin reconfiguration and binding of histone modifiers to actively transcribing genes. The 19S ATPase S7 was strongly associated with endogenous Ser5 phosphorylated-RNA Pol II (Figure 8). While 19S ATPase knockdown had no impact on global RNA Pol II levels, there was a specific decrease in levels of Ser5p-RNA Pol II in lysates from each of the ATPase knock down cells (Figure 9). Together these data suggest variable roles for the 19S ATPases in transcription initiation or in the transition of RNA Pol II to a productive elongation phase.

It has been proposed that the 19S ATPases participate in nucleosome eviction and chromatin remodeling necessary to release paused RNA Pol II [15], [43]. As the 19S ATPases associate with DNA, with elongation factors and with the promoter region of CIITApIV, we determined whether the 19S ATPases are involved in the recruitment of RNA Pol II to the CIITApIV promoter for phosphorylation and subsequent elongation. RNA Pol II and TBP binding to CIITApIV was significantly reduced in the absence of 19S ATPases (Figure 10) while mRNA levels were less impacted by 19S ATPase knockdown (Figure 3). The relative difference in impact on RNA Pol II recruitment versus mRNA expression suggests that low level recruitment of RNA Pol II to CIITApIV drives modest transcription of CIITA, albeit with greater difficulty the further down the CIITApIV gene the polymerase must transcribe. RNA Pol II overcomes nucleosome barriers with closely spaced transcribing RNA Pol II displacing core histones and promoting transcription. While the density of RNA Pol II molecules bound to CIITApIV affects rates of transcription, impaired transcription persists in the absence of 19S ATPases. Together, these studies indicate the 19S ATPases play roles in recruitment and/or processivity of RNA Pol II at CIITApIV genes. Indeed, recent studies of the holo RNA Pol II complex indicate the mediator complex contains the 19S ATPase Sug1 [60]; together with our findings, these data indicate multiple roles for the 19S ATPases in the regulation of transcription elongation.

Further delineation of the specific mechanisms by which individual 19S ATPases influence transcription will provide information about the complex role of the ATPases in the stages of transcription. As a part of the 26S proteasome complex, only the S6a ATPase binds the polyubiquitinated chains of substrates targeted for proteasome mediated degradation [61] and S6a alone binds the coding region of the human immunodeficiency virus type 1 gene in the presence of the transcription factor Tat [62]. During protein degradation, yeast 19S ATPases Rpt1, Rpt2, and Rpt6 (human S7, S4, and Sug1, respectively) provide the pulling force by which substrates unfold and are pulled inwards toward the 20S core while the remaining 19S ATPases, Rpt3, Rpt4, and Rpt5, (human S6b, S10b, S6a, respectively) stabilize the substrate and translocate it towards the 20S core for degradation [57]. Our data now indicates the S7 ATPase interacts strongly with CDK9 and with Ser5p RNApol II. Thus the possibility remains that despite the similar impact of knockdown of Sug1, S7, and S6a on transcription at CIITApIV, the 19S ATPases may have independent functions in transcription. In fact, only the 19S ATPase S6a has previously been shown to contain bona fide ATPase activity [63]; and it is the S6a ATPase motif, but not the S6a helicase motif, which is necessary to enhance transactivation of inducible genes [64]. It remains to be determined if a similar ATP driven unwindase activity, and potentially some unique activity of S6a and/or other 19S ATPases, is utilized to drive requisite conformational changes in histones, in transcription factors, and/or in coactivators to regulate their interactions with promoters and coding sequences and to allow transcription processes to occur.

## Supporting Information

Figure S1 **(A, B, C) siRNA Efficiency.** Expression of Sug1, S7 and S6a was specifically decreased using ATPase specific siRNA. Blots shown are indicative of data from three biologically independent experiments.
(TIFF)

Figure S2 **(A, B) siRNA Efficiency.** Sug1, S7, and S6a protein expression was effectively decreased using specific siRNA. Actin blots demonstrate loading and siRNA specificity controls. Blots shown are indicative of data from three biologically independent experiments.
(TIFF)

Figure S3 **(A, B, C) siRNA Efficiency.** Sug1, S7, and S6a protein expression was effectively decreased using ATPase specific siRNA. Blots shown are indicative of data from three biologically independent experiments.
(TIFF)

Figure S4 **(A, B, C) siRNA Efficiency.** Sug1, S7, and S6a protein expression was effectively decreased using ATPase specific siRNA. Blots shown are indicative of data from three biologically independent experiments.
(TIFF)
