# Supplementary figures and images for: Nonproteolytic Roles of 19S ATPases in Transcription of CIITApIV Genes
Source: PLoS One. 2014 Mar 13;9(3):e91200. doi: 10.1371/journal.pone.0091200 (PMC3953376; doi:10.1371/journal.pone.0091200)

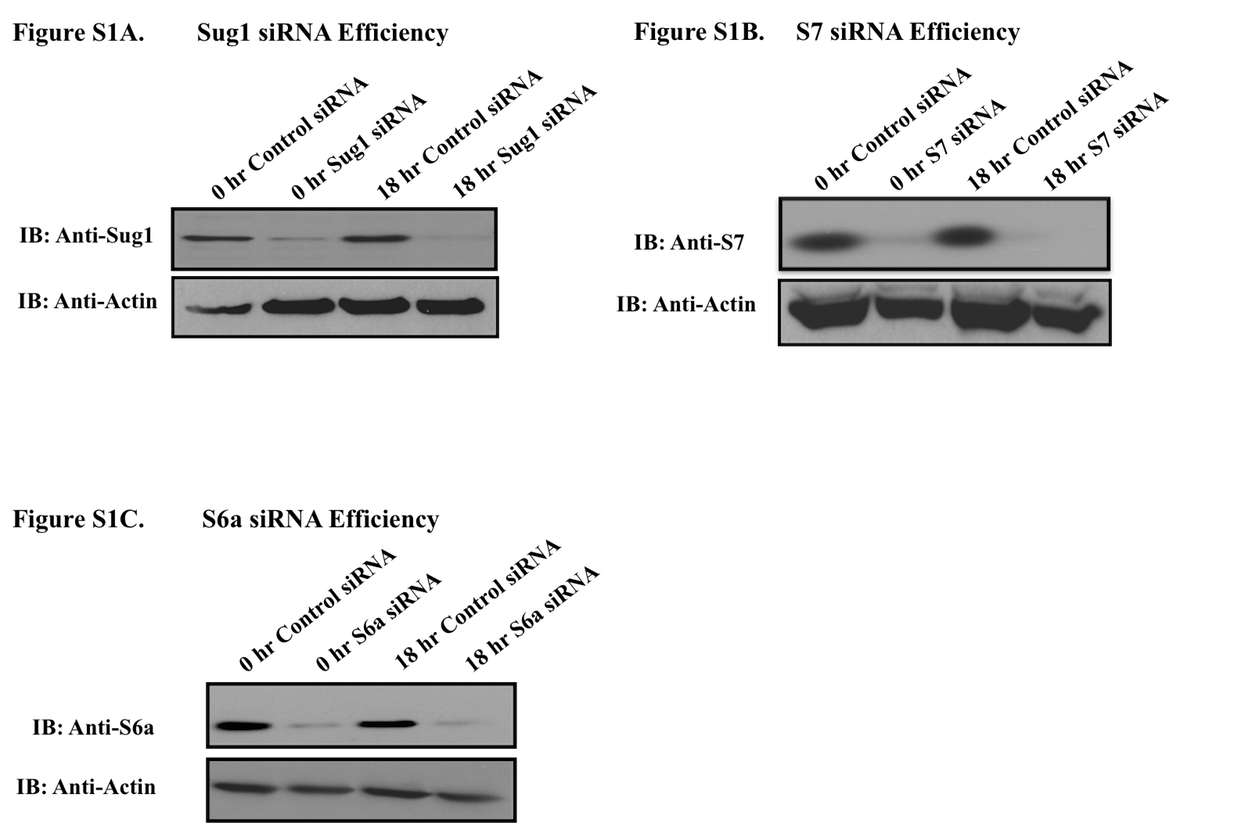

Supplement: Figure S1 — (A, B, C) siRNA Efficiency. Expression of Sug1, S7 and S6a was specifically decreased using ATPase specific siRNA. Blots shown are indicative of data from three biologically independent experiments. (TIFF) [file pone.0091200.s001.tiff]

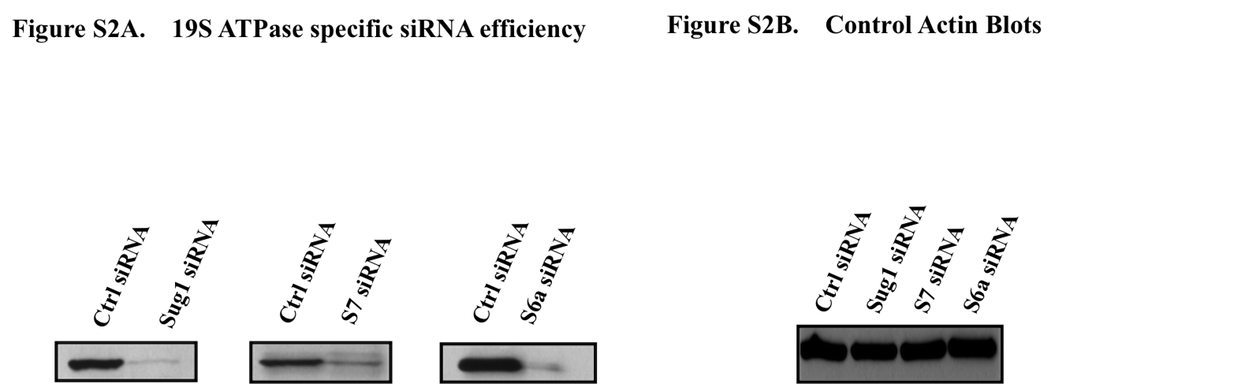

Supplement: Figure S2 — (A, B) siRNA Efficiency. Sug1, S7, and S6a protein expression was effectively decreased using specific siRNA. Actin blots demonstrate loading and siRNA specificity controls. Blots shown are indicative of data from three biologically independent experiments. (TIFF) [file pone.0091200.s002.tiff]

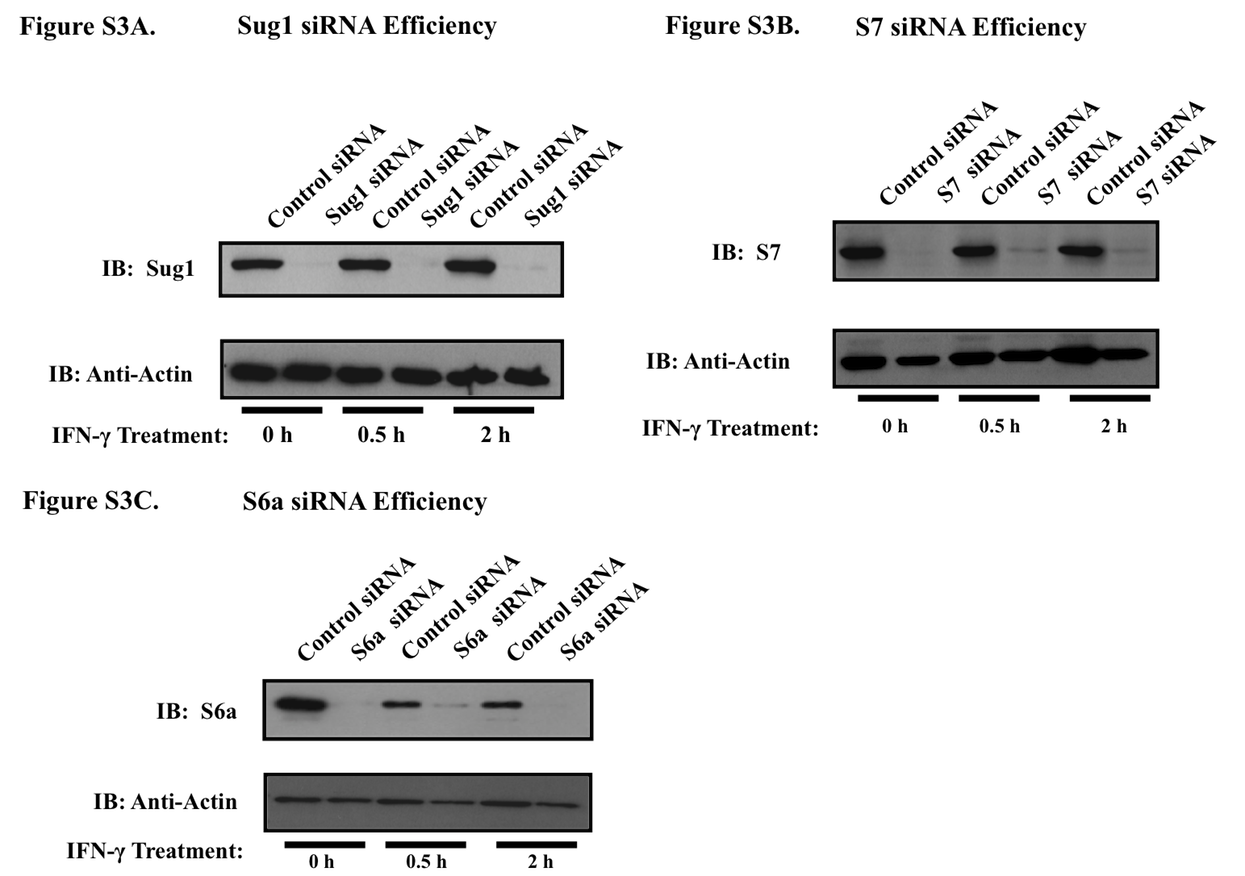

Supplement: Figure S3 — (A, B, C) siRNA Efficiency. Sug1, S7, and S6a protein expression was effectively decreased using ATPase specific siRNA. Blots shown are indicative of data from three biologically independent experiments. (TIFF) [file pone.0091200.s003.tiff]

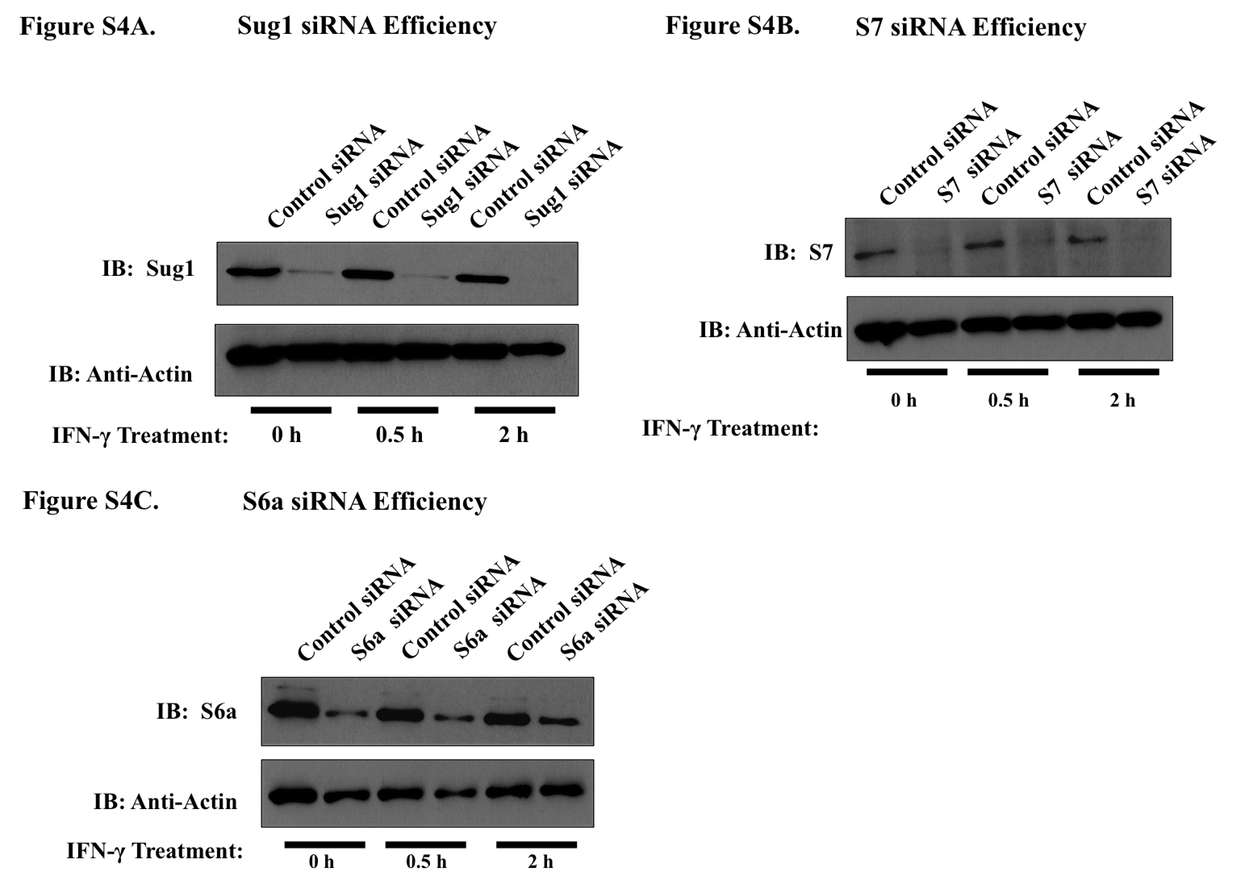

Supplement: Figure S4 — (A, B, C) siRNA Efficiency. Sug1, S7, and S6a protein expression was effectively decreased using ATPase specific siRNA. Blots shown are indicative of data from three biologically independent experiments. (TIFF) [file pone.0091200.s004.tiff]
